# Features of the First Case of Foodborne Botulism Caused by Dual-Toxin *Clostridium parabotulinum* Subtype A1(B5) in Spain

**DOI:** 10.3390/toxins17090429

**Published:** 2025-08-27

**Authors:** Sylvia Valdezate, Mónica Valiente, Gema Carrasco, María J. Medina-Pascual, María Isabel Hurtado, Maite Ruiz de Pipaón, Noelia Garrido, Carmen Paradas, José Ramón Hernández-Bello, Pilar Villalón

**Affiliations:** 1 Reference and Research Laboratory for Taxonomy, National Centre of Microbiology, Instituto de Salud Carlos III, 28220 Majadahonda, Spainpvillalon@isciii.es (P.V.); 2 Servicio de Microbiología, Hospital Virgen del Rocío, 41013 Sevilla, Spain; 3 CSUR/ERN de Enfermedades Neuromusculares, UGC Neurología, Hospital Universitario Virgen del Rocío/IBiS, 41013 Sevilla, Spain; 4 Sección Epidemiología, Servicio de Salud, Delegación Territorial de Salud y Consumo en Sevilla, 41018 Sevilla, Spain

**Keywords:** botulinum neurotoxin, BoNT/A(B), *Clostridium parabotulinum*, food-borne botulism

## Abstract

The neurotoxin BoNT/B2 is the predominant *Clostridium parabotulinum* subtype in foodborne and infant botulism cases in Spain. This study characterizes a novel case of foodborne botulism in Spain caused by a dual-toxin A1(B5) strain. A 64-year-old male presented with acute, progressive flaccid paralysis including diplopia, dysphagia, and respiratory failure. Although botulism was not initially suspected, the patient recovered with supportive care and without antitoxin administration. Genomic characterization confirmed the presence of both *bont*/A1 and silent *bont*/B5 genes. The *bont*/A1 gene was associated with an *orf*X+ neurotoxin gene cluster, while the silent *bont*/B5 gene was in an *ha*+ cluster. Phylogenetic analysis of both *bont*/A1 and *bont*/B5 sequences showed 100% amino acid identity, respectively, to previously reported A1(B5) strains (e.g., CDC_69094, FE9504ACG). Multi-locus sequence typing (MLST) assigned the ST10, a genotype previously undetected in Spanish botulism cases, yet found in other European countries. This case highlights the importance of considering botulism in differential diagnosis due to its varied presentation and the significance of timely laboratory confirmation for effective management. The identification of this dual-toxin BoNT/A1(B5) *orf*X+/*ha*+ ST10 strain expands our understanding of *C. botulinum* epidemiology and genetic diversity in Spain.

## 1. Introduction

Botulism is an infrequent, yet severe, neurotoxin-mediated disease characterized by flaccid descending paralysis. Without prompt intervention, it can progress to respiratory failure and death. Botulinum neurotoxins (BoNTs), the most potent natural toxins known, translocate into the nerve cell cytoplasm at neuromuscular synapses. There, they specifically cleave SNARE proteins of motor neurons, inhibiting the release of the neurotransmitter acetylcholine [1,2]. Neurotoxin exposure can occur through various routes, including ingestion (foodborne botulism), bacterial colonization of wounds (wound botulism) or the intestinal tract (infant botulism and adult intestinal colonization botulism), and iatrogenic administration of high-concentration cosmetic or therapeutic toxin injections. Foodborne botulism typically results from the ingestion of preformed neurotoxins produced by bacterial proliferation within contaminated food matrices [3,4].

BoNTs constitute a diverse group of zinc metalloproteases, classified into nine types (BoNT/A to BoNT/G, H or F/A, X) based on their antigenic activity. They are further subdivided into over 41 subtypes according to sequence variations (subtypes A1–A8, B1–B8, E1–E12, and F1–F8). BoNT subtypes differ in amino acid sequence from 7% (BoNT/B) to 36% (BoNT/F) and can be located on a plasmid or chromosome [5,6]. Although all neurotoxins induce similar clinical manifestations, variations in disease severity may be observed [1].

BoNTs are produced by seven distinct clostridial species, which are anaerobic, Gram-positive, spore-forming rods. The primary species is *Clostridium parabotulinum* or *C. botulinum* group I, a historical designation for proteolytic clostridia that produce BoNT/A, BoNT/B, or BoNT/F. The six additional genospecies include *Clostridium botulinum* (*C. botulinum* group II), *Clostridium novyi* sensu lato (*C. botulinum* group III), *Clostridium argentinense*, *Clostridium baratii*, *Clostridium butyricum*, and *Clostridium sporogenes*. Significant evidence supports the horizontal transfer of botulinum neurotoxin genes between distant related bacterial taxa [7,8,9].

While most clostridial strains produce a single BoNT type, some strains exhibit bivalency [Ab, Ba, Af, Bf (with lowercase letters denoting lesser production)] or trivalency (e.g., strain Af84, producing BoNT/A2, F4, and F5). In other cases, strains harbor silent neurotoxin genes due to mutations or truncations, thus not producing active toxin proteins [10]. For example, type A strains with silent *bont*/B [(*bont*/B)] genes are designated as type A(B) strains [11]. All *bont* genes are located within a neurotoxin gene cluster (on the chromosome, plasmids, or phages), arranged into two different conformations. One conformation includes hemagglutinin (HA) genes (*ha*17, *ha*33, and *ha*70), associated with *bont*/B, *bont*/C, *bont*/D, and *bont*/G. The other conformation contains *orf*X genes, encoding proteins of unknown function (*orf*X1, *orf*X2, and *orf*X3), linked to *bont*/A2, A3, A4, E, and F and a gene (p47) encoding a 47 kDa protein. The *bont*/A1 gene is the only one that can be present in either *ha* or *orf*X botulinum toxin loci. Immediately upstream of the *bont* gene is the *ntnh* gene, encoding the non-toxic non-hemagglutinin (NTNH) protein, which serves to protect BoNT from degradation [12,13].

According to the latest available ECDC report for 2022, 13 out of 30 EU/EEA countries reported 84 confirmed cases of botulism (notification rate of 0.02 cases/100,000 inhabitants). Italy, Romania, and France accounted for 68% of the reported cases. In 2021, Spain, along with Italy and Romania, accounted for 66% of cases. The 2022 distribution of BoNT types in EU countries was as follows: BoNT/B (61 cases; 85%), BoNT/A (8 cases; 11%, including two cases with concurrent BoNT/B and one with BoNT/E), BoNT/E (2 cases), and BoNT/F (1 case). Dual-toxin botulism is a rare occurrence in the EU, with no cases reported in the preceding three years [14]. Since 2010, Spain has shown a clear predominance of BoNT/B (specifically subtype BoNT/B2) in human botulism cases, with sporadic detections of subtypes BoNT/A (BoNT/A1-A2) and BoNT/F (BoNT/F7-F8) [15].

This report presents, for the first time in our country, a case of foodborne botulism caused by a dual-toxin gene strain A(B).

## 2. Results

### 2.1. Case Presentation and Management

In 2024, a 64-year-old male patient presented with one day of diplopia, ptosis, dysphagia and paresthesia in the right hand and leg. The following day, he developed worsening dysphagia and dysphonia, leading to hospital admission. Neurological examination revealed palpebral, vocal neck flexor fatigability, along with fatigability in the upper limbs. The weakness rapidly progressed, resulting in complete eyelid closure, severe neck muscle weakness, and unresponsive respiratory failure, requiring endotracheal intubation and ICU admission. The initial diagnosis was a probable generalized myasthenia gravis with bulbar predominance. Treatment was initiated with prednisone (1 mg/kg) and intravenous immunoglobulins (2 g/kg). The patient’s medical history included a diffuse large B-cell lymphoma, germinal center phenotype stage IV-Xe-B, diagnosed 10 years prior, which achieved complete remission after six cycles of R-CHOP chemotherapy (rituximab, cyclophosphamide, doxorubicin hydrochloride, vincristine sulfate, and prednisone). Initial blood tests were unremarkable. Supplemental Figure S1 shows the patient’s clinical flowchart.

A detailed neurological examination showed normal pupils and ocular movements, but revealed bilateral ptosis, severe facial and lingual palsy, anarthria, and head drop. Manual muscle testing demonstrated symmetric weakness in upper limbs (arm abduction 2/5; arm extension and flexion 5/5; extensor digitorum and interosseous muscles 3/5) and lower limbs (hip flexion 0/5; all other muscles 5/5). Deep tendon reflexes were weakly evoked, with absent ankle reflexes. Cognitive functions and sensory remained spared. Tests for anti-acetylcholine receptor, gangliosides, P/Q-type voltage-gated calcium channel, and neuronal antibodies were all negative. Lumbar puncture and cranial tomography were normal.

Prednisone was discontinued on day 20 of illness. The patient’s condition began to slowly improve from day 25 following symptom onset. Subsequently, during a neurological review, a diagnosis of botulism was reconsidered due to the remarkably abrupt clinical presentation, which included diplopia, dysphagia, rapid progression to respiratory failure, and facial diplegia within a few days. These clinical findings, coupled with electromyography (EMG) results, further supported the suspicion. EMG showed generalized and notable reduced compound muscle action potential amplitudes in all the examined nerves of the upper and lower limbs. Repetitive stimulation at 3 Hz and 40 Hz did not show decrement or facilitation, respectively. Single-fiber EMG study was normal.

Botulinum antitoxin was not administered given the delay between symptom onset and clinical suspicion. The patient’s clinical course was favorable with supportive medical treatment, and his health condition resolved within two months. Epidemiological surveillance of food consumption failed to ascertain the source of acquisition.

### 2.2. Laboratory Diagnosis and Confirmation of a Dual-Toxin C. parabotulinum Strain

Standard mouse bioassay performed on patient stool samples collected on day 25 of illness yielded negative results. However, multiplex PCR confirmed the presence of both *bont*/A and *bont*/B genes [16,17]. Subtyping identified these as BoNT/A1 and BoNT/B5, respectively, based on their defined representative subtypes. The BoNT/A1 sequence of the present case showed the changes 2-Pro → Gln and 27-Ala → Val, when compared with the representative strain ATCC 3502 (1296 amino acids, GenBank accession no. CAL82360). The BoNT/B5 subtype in this case exhibited the following amino acid substitutions: 18-Asn → Ile, 47-Pro → Leu, 71-Cys → Trp and 128-Glu → stop codon, with respect to the representative strain Ba4 657 (1291 amino acids, GenBank accession no. ACQ51206), resulting in a silent neurotoxin.

**Figure 1:**
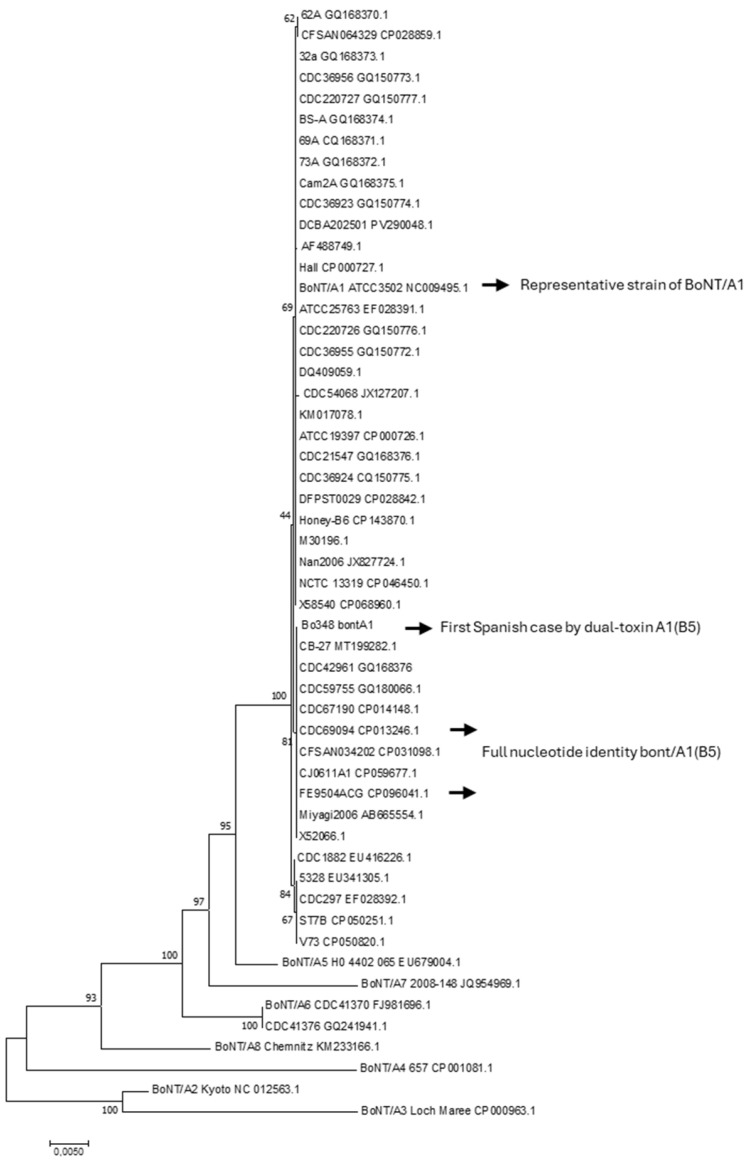
Maximum likelihood (ML) phylogenies of *bont*/A1 nucleotide sequences (*n* = 45) available in NCBI, along with representative sequences from BoNT/A1–A8 subtypes. The Spanish case is identified as Bo348. Each sequence was coded by its strain identification number followed by its sequence identification number. The reliability of the ML topologies was assessed via 1000 bootstrap replications with the Tamura–Nei model. The percentage of trees on which the associated taxa clustered together is indicated next to the branches. The tree is drawn to scale, with branch lengths representing the number of substitutions per site. Phylogenetic analyzes were conducted using the MEGA7 software [18].

**Figure 2:**
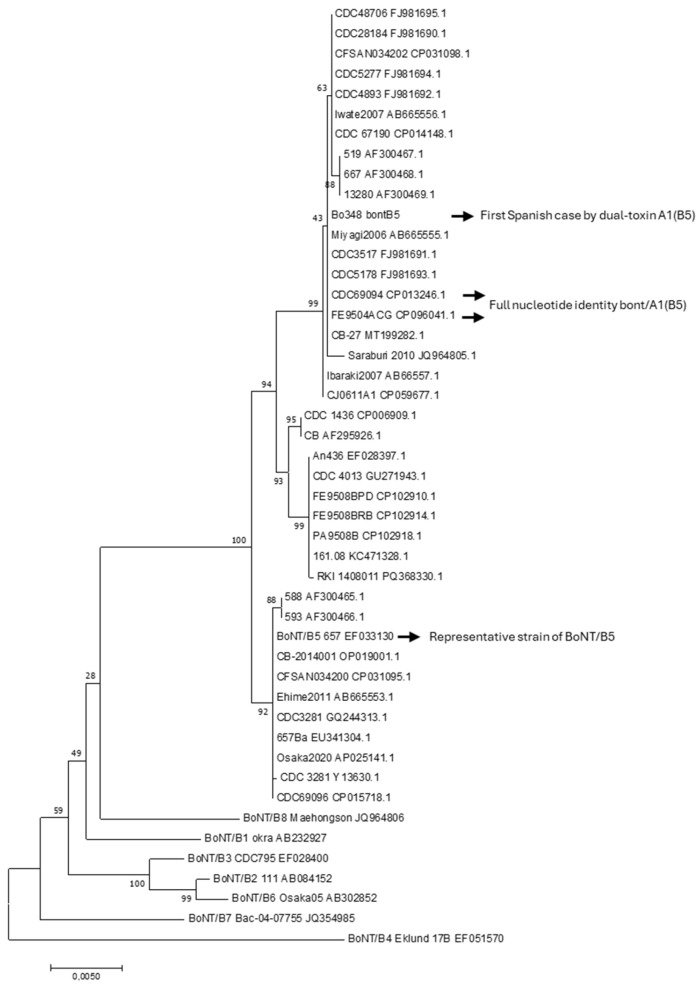
Maximum likelihood (ML) phylogenies of *bont*/B5 nucleotide sequences (*n* = 39) available in NCBI, along with representative sequences from BoNTB1–B8 subtypes. The Spanish case is identified as Bo348. Each sequence was coded by its strain identification number followed by its sequence identification number. The reliability of the ML topologies was assessed via 1000 bootstrap replications with the Tamura–Nei model. The percentage of trees on which the associated taxa clustered together is indicated next to the branches. The tree is drawn to scale, with branch lengths representing the number of substitutions per site. Phylogenetic analyzes were conducted using the MEGA7 software [18].

Maximum likelihood (ML) phylogenies of the *bont*/A1 and *bont*/B5 genes, including the available nucleotide sequences from the NCBI database and representative sequences of BoNT/A and BoNT/B subtypes, were constructed (Figure 1 and Figure 2) [18].

For the group of strains analyzed, *bont*/A1 nucleotide sequences exhibited less diversity than *bon*t/B5 sequences. The dissimilarity percentage among the different *bont*/A1 and *bont*/B5 sequences ranged from 0.1 to 0.2% and 0.1 to 0.7%, respectively. BLASTn/BLASTp analysis of the studied case’s sequences revealed 99.97% and 100% nucleotide sequence identity for *bont*/A1 and *bont*/B5, respectively, when compared with the corresponding bivalent A1(B5) strains CDC_69094 (CP013246.1) and FE9504ACG (CP096041) [19,20,21].

The protein sequences for BoNT/A1 and BoNT/(B5) from these strains were identical with those of the Spanish case. In contrast, this case demonstrated the same amino acid substitutions (2-Gln and 27-Val), as noted previously, when compared with other BoNT/A1 cases (*n* = 3) attributed to monovalent strains reported in our country.

Analysis of the non-toxic non-hemagglutinin gene (*ntnh*), located upstream of the botulinum neurotoxin gene in all known clusters, differentiated two amplicon sizes: 225 bp for ntnh genes associated with *orf*X+ toxin gene clusters, and 325 bp for *ntnh* genes associated with *ha*+ toxin gene clusters. Consistent with previous findings, the *bont*/A1 gene of this case is located within an *orf*X+ type neurotoxin gene cluster, while the silent *bont*/B5 gene is located on a *ha*+ type gene cluster [22]. This arrangement differs from previous monovalent toxin BoNT/A1 cases in our country, where the *bont*/A1 gene has typically been located on a *ha*+ type cluster.

### 2.3. Genetic Relatedness by Flagellin A and Multi-Locus Sequence Type

**Figure 3:**
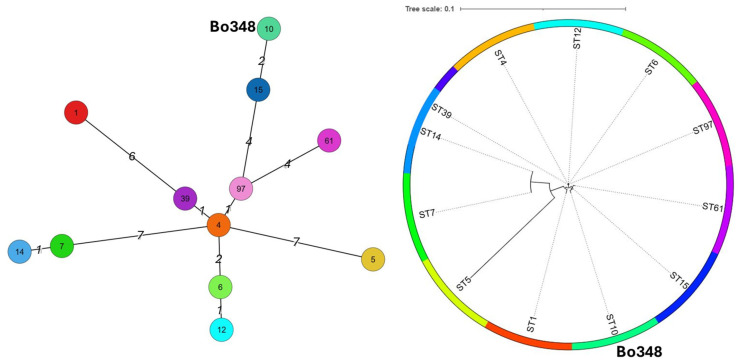
Phylogenetic relationships of the ST10 from the Spanish case study with other STs identified in *C. parabotulinum* BoNT/A(B) strains available on PubMLST (https://pubmlst.org/organisms/clostridium-botulinum, accessed on 11 April 2025) [25]. Trees were constructed using two routines: In the left panel, GrapeTree was used to create a minimum spanning tree from allelic profiles [26]; the number of allelic differences is indicated in italics. In the right panel, iTOL (https://itol.embl.de/, accessed on 27 July 2025) was used to generate a neighbor-joining tree from the concatenated nucleotide sequences [27].

The dual-toxin A1(B5) of the Spanish case exhibited flagellar type 1, the most frequent type found in *C. parabotulinum* [23]. Multi-locus sequence typing (MLST) assigned the sequence type ST10, with the following gene–allele numbers: *aro*E-11, *mdh*-5, *ace*K-11, *opp*B-5, *rpo*B-8, *rec*A-6, and *hsp*60-8. The PubMLST database identified twelve distinct STs for A(B) strains (*n* = 46): ST1, ST4, ST5, ST6, ST7, ST10, ST12, ST14, ST15, ST39, ST61, and ST97 [24]. Among these, ST4 and ST7 were the most predominant. Strains CDC_69094 (CP013246.1) and FE9504ACG (CP096041) were both classified as ST6 [19,20,21]. The ST6 designation is based on the following allelic profile: *aro*E-9, *mdh*-10, *ace*K-6, *opp*B-9, *rpo*B-6, *rec*A-7, and *hsp*60-7. Notably, this ST6 profile shows seven out of seven allelic differences when compared with the ST10 exhibited by the studied case. Genetic relationships among these STs are illustrated in Figure 3, generated using PubMLST routines such as iTol and GrapeTree [25,26,27].

**Figure 4:**
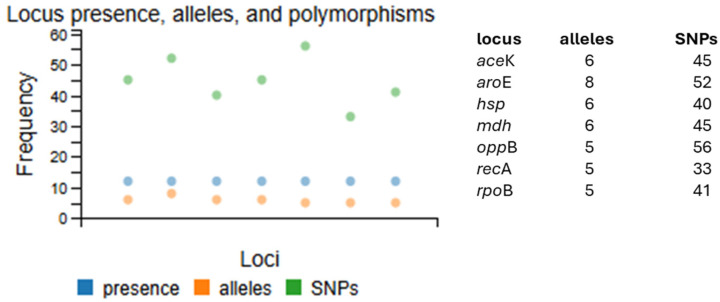
MLST Loci, presence, alleles, and polymorphism in *C. parabotulinum* BoNT/A(B) strains available on PubMLST (https://pubmlst.org/organisms/clostridium-botulinum, accessed on 16 April 2025) [25]. The x-axis represents the genes of the scheme.

The diversity (loci, allele presence, and polymorphism) observed for the genes integrated into the MLST scheme for *C. parabotulinum* BoNT/A(B) strains available on the snp-sites tool of PubMLST (https://pubmlst.org/organisms/clostridium-botulinum, accessed on 16 April 2025) [25] is illustrated in Figure 4. Each locus exhibited a high degree of polymorphism (range of 33–56 sites, average 44.5), resulting in up to five, six, and eight alleles for the MLST genes.

## 3. Discussion

This report provides insights into the clinical and microbiological presentation of the first documented case of botulism caused by a dual-neurotoxin-producing *C. parabotulinum* strain, specifically subtype A1(B5), in Spain. Botulism is a neurological condition that is frequently delayed or even missed [28]. It is commonly misdiagnosed as other neurological conditions, such as myasthenia gravis and Guillain–Barré syndrome, with a wide range of common and unusual etiologies included in the differential diagnosis (e.g., cerebrovascular accident, Lambert–Eaton syndrome, meningitis, encephalitis, and tick paralysis) [1,16]. Although these conditions can present with similar symptoms, particularly in the early stages of intoxication, specific clinical clues aid in their differentiation. For instance, in botulism, pupils often become dilated with a sluggish or absent light reflex, whereas the pupils are typically unaffected in myasthenia gravis [29]. The most reported symptoms among botulism patients include dysphagia, blurred vision, slurred speech, difficulty speaking, hoarse voice, gastrointestinal symptoms, dry mouth, shortness of breath, and diplopia. Key signs typically include descending paralysis, ptosis, and ophthalmoplegia. Botulism characteristically presents with a distinctive syndrome of cranial nerve palsies, often followed by bilateral, symmetric, descending flaccid paralysis that affects proximal before distal limb musculature, which can progress to respiratory failure and death [1,4].

The latency period of botulism toxidrome varies depending on patient factors and the specific type and quantity of ingested toxin. Symptoms typically appear within 12 to 36–48 h after consuming contaminated food. However, symptom onset can be delayed in some instances for up to 10–15 days. This variability in onset time can complicate accurate diagnosis, especially in cases like the present one, where the food source could not be identified [30].

On-time diagnosis is critical for effective treatment and preventing fatal outcomes because botulinum antitoxin heptavalent (BAT), the specific therapy for botulism, must be administered early in the course of illness. While clinical symptoms and patient history are vital, a positive laboratory diagnosis should always be sought. Clinical guidelines therefore recommend considering botulism in the presence of this spectrum of signs and symptoms, ranging from limited cranial nerve palsies (e.g., ptosis) to respiratory failure and complete extremity paralysis, without necessarily waiting for laboratory confirmation. Differential diagnosis is more frequently required for sporadic or isolated cases, whereas the observation of multiple linked cases, as commonly occurs with foodborne botulism, often makes diagnosis more straightforward [1,4,29,30]. Furthermore, treatment should not be unduly delayed while awaiting laboratory confirmation. Laboratory results can take several days due to pre-enrichment and culture requirements, and delaying antitoxin administration in a patient with a high or medium likelihood of botulism while awaiting results can significantly worsen the patient’s outcome. In fact, delays in administering antitoxin to positive cases are consistently associated with increased morbidity [29]. Consequently, the diagnosis of botulism relies heavily on a high clinical suspicion and a thorough neurological examination.

Despite its critical role, serum-based antitoxin treatment has considerable limitations. It is unable to neutralize botulinum toxin once it has been internalized by motor neurons. The antitoxin functions by binding to and neutralizing circulating toxin solely within the bloodstream by blocking the heavy-chain receptor’s attachment to presynaptic receptors. This mechanism leads to the clearance of the toxin–antitoxin complex from circulation [31]. Studies indicate that patients who receive botulinum antitoxin within 48 h of symptom onset typically experience shorter hospital stays and require less intensive care [29]. Therefore, clinicians are strongly advised to initiate treatment for all suspected botulism cases without delay, rather than waiting for laboratory confirmation, as this minimizes toxin internalization and counters the rapid, irreversible progression of the intoxication. For successful laboratory confirmation of botulism, specimens must be collected as soon as a case is suspected. This is crucial because toxin levels in both serum and stool samples rapidly decrease over time as the toxin is internalized into neurons. Any delay in sample collection, or storage above refrigerator temperature (2–8 °C), can lead to false-negative results. It is important to note that serum samples must be collected prior to the administration of the botulism antitoxin. However, stool samples may be collected post-treatment since *Clostridium* species are not affected by the antitoxin. Regardless, all samples should be collected before antibiotic therapy begins [4,29].

Structurally, botulinum neurotoxins are zinc metalloproteases, each comprising a 50 kDa catalytic light chain (LC) disulfide bonded to a 100 kDa heavy chain. Synaptic transmission is transiently and reversibly inhibited when the BoNT LCs cleave specific target proteins—namely the synaptosomal-associated protein of 25 kDa (SNAP25), the vesicle-associated membrane protein (VAMP), and syntaxin—within the presynaptic termini [13,32]. Despite these similar mechanisms of action, the duration of muscle paralysis varies considerably depending on the type. For example, while both BoNT/A and BoNT/E target SNAP25, paralysis induced by BoNT/A can endure for several months, whereas the effects of BoNT/E are comparatively brief [33]. Therefore, recovery from botulism can range from weeks to several months. Its duration depends not only on the type, but also on the quantity of the ingested botulinum neurotoxin. Prompt treatment and antitoxin administration can often lead to full recovery within two weeks without residual symptoms [29]. The recovery observed in our case study, involving dual-toxin A1(B5), took two months.

The underlying mechanism of recovery from intoxication and the restoration of neurotransmitter release involves the natural protein turnover leading to the degradation of BoNT within neuronal termini, coupled with the regeneration of nerve endings from affected neurons [33]. It is hypothesized that the differential toxicity and persistence observed among toxin types correlate with their varying resistance to the ubiquitin–proteasome system (UPS)-mediated protein turnover. Consequently, novel therapeutic strategies are being investigated to accelerate botulism recovery times by enhancing the turnover of internalized BoNT [34].

Foodborne botulism, a severe and often lethal neuroparalytic intoxication, can be triggered by the consumption of as little as 50 ng of botulinum neurotoxin (BoNT). This condition is primarily caused by *C. parabotulinum* group I strains, and to a lesser extent, *C. sporogenes*. The spores of *C. parabotulinum* group I are the target of the “botulinum cook” (121 °C/3 min), a critical thermal process applied to low-acid canned foods. Historically, outbreaks of foodborne botulism have been linked to failures in properly applying the botulinum cook to canned or bottled foods, as well as to the temperature abuse of products intended for chilled storage. Given the significant public health and commercial implications of these outbreaks, extreme vigilance remains essential to minimize the incidence of foodborne botulism [8,35,36].

In the current Spanish case, a stool sample collected one month after symptom onset showed positive results with two bands of amplification for *bont* and *ntnh* gene detection in cultures. However, the bioassay, which detects circulating neurotoxins, was negative due to late sample collection. This finding raises the possibility of transient adult intestinal colonization [37]. It is important to consider that the absence of circulating toxins does not always rule out botulism, especially when samples are collected late. The successful genetic detection in this present case, even with a negative bioassay, could be particularly relevant given that neurotoxin type A produces the most severe and often more persistent syndrome [38].

Significantly, neither the BoNT/B5 subtype nor the dual-toxin BoNT/A(B) with the silent *bont*/B5 gene had been previously identified in our country. The BoNT/B2 subtype is the primary cause of human botulism in Spain, with foodborne acquisition linked to multiple lineages [15]. This clear predominance of the BoNT/B2 subtype may be related to its widespread spore contamination of agricultural and food environments. This correlates with a recent survey conducted in France, which reported high rates of *C. parabotulinum* spores in soil, reaching values of 45.3%. While other countries show varying prevalence (5.7% in the United Kingdom, 16.5% in Japan, near to values of 23.0% in some American countries). The overall pattern, especially the high French figure, suggests a significant presence of *C. parabotulinum*, predominantly the BoNT/B type, in the environment [39].

While monovalent neurotoxins are common causes of foodborne botulism, the co-occurrence of expressed type A botulinum neurotoxin (BoNT/A) and unexpressed type B neurotoxin (BoNT/B) in foodborne outbreaks has been well-documented [1,40,41,42,43,44,45]. The comprehensive investigation of type A(B) strains, isolated from diverse sources across distinct foodborne botulism outbreaks in the USA, revealed substantial genomic sequence homology, with most of these strains clustering closely within the country. Specifically, these strains frequently exhibited ST4 [44]. Recently, genomic analysis of BoNT/A1(B) strains identified two distinct, conserved clades that displayed geographically restricted distributions. All but one of the strains within these clades were found to originate from the Northern Hemisphere. The larger, predominant clade was chiefly concentrated in the USA, whereas the smaller clade comprised genomes from Italy and Japan [46].

A broader search of the PubMLST database revealed that only 6% (12 out of 193 described STs) are associated with botulism caused by A(B) strains. These A(B) STs are distributed worldwide [30,44,45,46]. Yet, they display high inter-ST diversity (Figure 3 and Figure 4). Interestingly, some previously characterized A1(B5) strains, such as CDC_69094 (CP013246.1) and FE9504ACG (CP096041), showed complete identity in their *bont*/B5 sequences and high identity in their *bont*/A1 sequences when compared with the corresponding sequences of the current case [19,20,21]. These strains were isolated from a patient’s stool during a foodborne outbreak investigation in the USA and from an infant botulism case in Canada, respectively. Both strains, despite their different origins, are classified as ST6, making them genetically very distinct from ST10. Notably, the ST10 observed in the present case had not been previously reported in botulism events in Spain, with other STs previously involved in BoNT/A strains in the country [15]. It is worth noting that ST10 has been detected in three other types of BoNT/A strains globally, two of which were isolated in France and Morrocco in 2007 and 2012 [43]. These findings underscored the considerable genomic diversity within *C. parabotulinum* BoNT/A(B) strains at a global level.

## 4. Conclusions

This case highlights the ongoing challenges in diagnosing and managing botulism, particularly with rare dual-toxin strains and atypical presentations. Therefore, we emphasize the urgent need for enhanced clinician awareness to ensure timely and accurate diagnosis and treatment. The identification of an A1(B5) *orf*X+/*ha*+ ST10 strain in Spain, underscores the dynamic epidemiology of *C. parabotulinum* and the importance of robust, international integrated microbiological surveillance systems. Such systems, combining clinical suspicion with comprehensive laboratory and molecular analyzes like MLST, are crucial for botulism diagnosis and the early detection of outbreaks, swift source identification, and effective public health interventions to minimize botulism incidence and prevent severe outcomes. Submitting the features of strains involved in botulism events to the PubMLST database enables the global tracking of sequence types and the detection of emerging clonal lineages. Prompt diagnosis and the on-time initiation of BoNT antiserum are crucial for limiting paralysis and preventing respiratory compromise, which remain pressing challenges in this toxin-mediated illness.

## 5. Material and Methods

### 5.1. Botulism Diagnosis

Stool sample was collected on day 20 after illness onset and submitted to the National Centre of Microbiology (Madrid, Spain) for botulism diagnosis. Standard mousse bioassay was performed in accordance with institutional animal care guidelines and regional authorization, using heptavalent anti-BoNT antiserum (BAT^®^, Emergent BioSolutions Canada Inc., Winnipeg, MB, Canada) [16,47]. To detect *bont* genes, multiplex PCR was carried out on DNA extracted from stool enrichment cultures [17].

### 5.2. Botulism Confirmation by Bont, flaVR and MLST Genes

For subtype identification, the complete *bont* genes were further characterized, and alleles were determined by comparison to defined representative strains for each subtype [5,6]. Identities relative to *bont*/A1 and *bont*/B5 available nucleotide sequences in the GenBank database (*n* = 45 and 39 sequences, respectively), were obtained using the NCBI BLASTn/BLASTp tool (https://blast.ncbi.nlm.nih.gov/Blast.cgi, accessed on 17 July 2025). Phylogenetic analyzes were conducted by Maximum likelihood (ML) phylogenies of the *bont*/A1 and *bont*/B5 genes. For alignment, the *bont*/A1 and *bont*/B5 sequences were adjusted at 3891 bp and 3437 bp, respectively. The representative nucleotide sequences of subtypes BoNT/A1-A8 and BoNT/B1-B8 were included [6]. The reliability of the ML topologies was assessed via 1000 bootstrap replications with the Tamura–Nei model using the MEGA7 software [18].

To differentiate between the two botulinum neurotoxin gene clusters, *orf*X+ or *ha*+, the non-toxic non-hemagglutinin gene (*ntnh*) was studied [22]. The variable region of flagellin A (*fla*VR) was characterized as previously described [23]. The multi-locus sequence typing (MLST) with a seven-loci scheme was applied, being assigned by the PubMLST database (https://pubmlst.org/organisms/clostridium-botulinum, accessed on 15 April 2025) [24,25]. Genetic relationships with respect to the sequence types (STs) displayed by other A(B) strains were analyzed.

## Data Availability

The BoNT/A1(B5) sequences were deposited in GenBank under the accession numbers PV933012- PV933013. Data about the microbiological characteristics have been submitted with isolate no. id.340 (ST10) to the PubMLST database of *Clostridium botulinum* (https://pubmlst.org/organisms/clostridium-botulinum, accessed on 15 July 2025).

## Supplementary Materials

The following supporting information can be downloaded at: https://www.mdpi.com/article/10.3390/toxins17090429/s1, Figure S1: First foodborne botulism case by dual A1(B5) strain in Spain flowchart.

## Abbreviations

The following abbreviations are used in this manuscript:

BoNT	Botulinum neurotoxin
ST	Sequence type
