# Supplementary figures and images for: Features of the First Case of Foodborne Botulism Caused by Dual-Toxin Clostridium parabotulinum Subtype A1(B5) in Spain
Source: Toxins (Basel). 2025 Aug 27;17(9):429. doi: 10.3390/toxins17090429 (PMC12473986; doi:10.3390/toxins17090429)

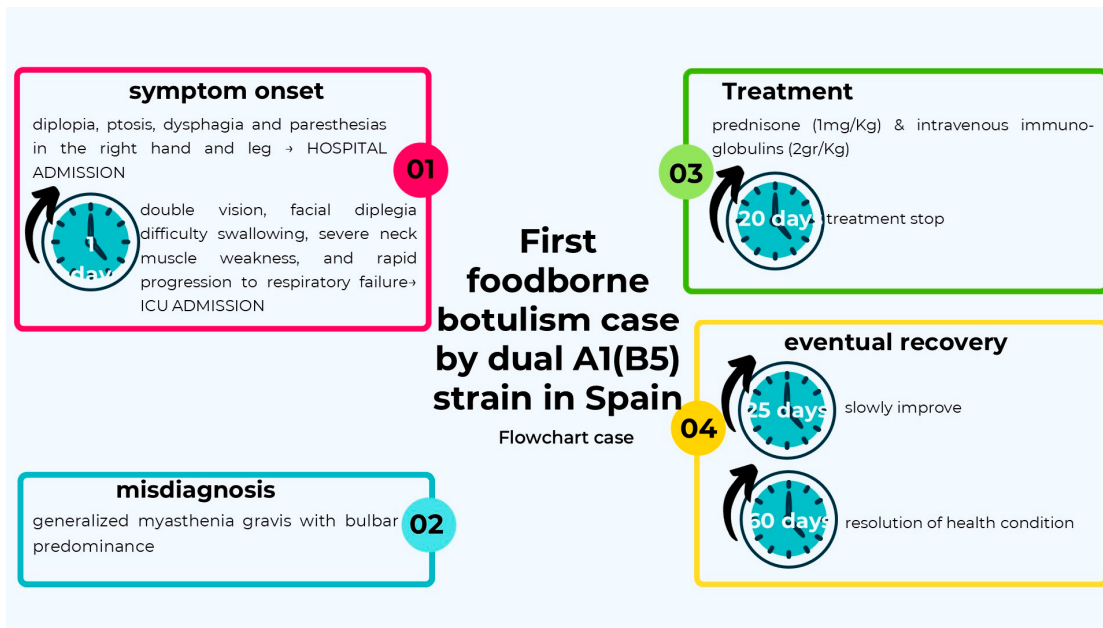

Figure S1. First foodborne botulism case by dual A1(B5) strain in Spain flowchart

Supplement: Supplementary file 1 [file toxins-17-00429-s001.zip › toxins-3814459-supplementary.pdf]
